# An Unusual Case of an Acute Episode of Restless Leg Syndrome Following Oral Metoclopramide Therapy

**DOI:** 10.7759/cureus.53754

**Published:** 2024-02-07

**Authors:** Mohammed A Aljunaid

**Affiliations:** 1 Department of Family and Community Medicine, University of Jeddah, Jeddah, SAU

**Keywords:** side-effects, semaglutide, vomiting, nausea, metoclopramide, restless leg syndrome

## Abstract

Restless leg syndrome (RLS) is a chronic disorder characterized by a compulsive urge to move the legs, accompanied by various subjective symptoms and a distinctive nyctimeral pattern. A negligent entity is drug-induced RLS, which may be challenging to recognize by practitioners due to its rarity. Among various drugs that can induce or exacerbate RLS, metoclopramide is notable; however, the literature primarily describes cases related to its intravenous forms. In this case presentation, a 33-year-old male experienced drug-related gastrointestinal (GI) symptoms after starting semaglutide for weight loss. Semaglutide was discontinued, and oral metoclopramide was administered to manage the GI symptoms. Subsequently, he developed RLS-like symptoms, which resolved within 48 hours of stopping metoclopramide. His family history included chronic RLS. Laboratory tests were normal. The case highlights a potential link between drug administration and transient RLS symptoms. This case suggests that RLS can be a rare, reversible side effect of oral metoclopramide. It emphasizes the need for careful monitoring of RLS symptoms in patients using this drug and highlights the variability of side effects depending on the method of drug administration. The case serves as a reminder of the unpredictable nature of drug reactions and the importance of vigilance in pharmacotherapy.

## Introduction

Restless leg syndrome (RLS) is a common chronic disorder primarily affecting the lower extremities. It is characterized by an irresistible urge to move the legs. Symptoms typically intensify during rest in the evening and diminish or cease when the patient begins walking or moving their legs [1]. The pathophysiology of this condition is not well understood. However, evidence suggests the role of iron deficiency in the onset and progression of RLS, likely by promoting dysfunction in the dopaminergic and serotoninergic systems in the brain and spinal cord. Additionally, RLS prevalence increases in patients with chronic renal failure, anemia, and pregnancy [2].

Drugs interfering with the dopaminergic system were shown to influence the development and severity of RLS [3]. Metoclopramide is a strong dopaminergic antagonist that acts on the D2-subtype dopamine receptor (D2R). It was approved by the FDA for the management of nausea and vomiting in patients with gastroesophageal reflux disease or diabetic gastroparesis. It is also used for chemotherapy-induced vomiting [4].

The occurrence or exacerbation of RLS has been described as a known side effect of intravenous (IV) metoclopramide [5]. Additionally, long-term use of metoclopramide may lead to movement disorders such as tardive dyskinesia and Parkinsonism, while dystonia and akathisia can potentially develop even after a single dose [6]. However, RLS is rarely described following oral metoclopramide.

This case report describes a male patient who developed acute symptoms of RLS following the administration of a second dose of oral metoclopramide, which was prescribed to manage semaglutide side effects.

## Case presentation

A 33-year-old male presented to our clinic with severe nausea and vomiting that persisted for two days. In the week prior to the onset of symptoms, the patient started taking semaglutide 0.25 mg for the first time as a weight-loss medication. On presentation, there were no other complaints besides the above-mentioned, and abdominal and neurological examinations were unremarkable. Due to the context and severity of the gastrointestinal symptomatology, a drug adverse effect was suspected; therefore, the patient was advised to discontinue semaglutide.

Before discharge, oral metoclopramide therapy (10 mg, twice daily) was prescribed to alleviate vomiting and nausea. However, after the third dose of metoclopramide, the patient experienced an unusual sensation in his legs, described as an uncontrollable urge to move his legs, accompanied by painful and unsettled "creepy crawly" sensations, predominantly during rest. Notably, vigorous leg movements provided temporary relief from the experienced symptoms, while movement cessation retriggered them. The patient reported no associated symptoms of anxiety or restlessness.

Importantly, the patient’s family history was remarkable for chronic RLS in one brother without any precise triggers or etiology. An extensive laboratory workup including a complete blood count as well as an ionic, renal, hepatic, metabolic, and hormonal profile revealed no pathological values (Table 1). There was no history of iron-deficiency anemia or chronic renal disease.

**Table 1:** Patient’s clinical and biological characteristics BMI: body mass index, MCV: mean corpuscular volume, MCH: mean corpuscular hemoglobin, TLC: total leucocyte count, CRP: C-reactive protein, HbA1c: glycated hemoglobin, BUN: blood urea nitrogen, eGFR: estimated glomerular filtration rate, ALT: alanine aminotransferase, AST: aspartate aminotransferase, GGT: gamma-glutamyl transferase, TSH: thyroid-stimulating hormone, LDH: lactate dehydrogenase, kg/m^2^: kilogram per square meter, bpm: beats per minute, mmHg: millimeters of mercury, g/dL: grams per deciliter, fL: femtoliters, pg: picograms, mg/dl: milligrams per deciliter, mmoL/L: millimoles per liter, U/L: units per liter, pg/mL: picograms per milliliter, ng/dL: nanograms per deciliter, uIU/mL: micro-international units per milliliter, ug/dL: micrograms per deciliter, ng/mL: nanograms per milliliter

Sex	Male
Age	33
BMI	29 kg/m^2^
Heart rate	78 bpm
Oxygen saturation	100%
Blood pressure	124/79 mmHg
Hemoglobin	14.3 g/dL
Hematocrit	42.9%
MCV	86.0 fL
MCH	28.7 pg
TLC	4.63 x10^9/L
Platelet count	233 x10^9/L
CRP	0.44 mg/dl
HbA1c	5%
BUN	15 mg/dL
Creatinine in serum	0.95 mg/dL
Uric acid in serum	5.4 mg/dL
Chloride in serum	106 mmoL/L
Potassium in serum	5.0 mmoL/L
Sodium in serum	142.0 mmoL/L
Calcium in serum (total)	9.4 mg/dL
Magnesium in serum	1.9 mg/dL
BUN/creatinine ratio	15.8
Globulin in serum	2.2 g/dL
eGFR	>60 mL/min/1.73 m^2^
Bilirubin (total)	0.31 mg/dL
Bilirubin (direct)	0.15 mg/dL
ALT	16 U/L
AST	19 U/L
Alkaline phosphatase	79 U/L
GGT	23 U/L
Protein in serum (total)	6.7 g/dL
Free T3	2.70 pg/mL
Free T4	0.92 ng/dL
TSH	0.50 uIU/mL
LDH	148 U/L
Cholesterol	155 mg/dL
Triglycerides in serum	116 mg/dL
HDL cholesterol	44 mg/dL
LDL cholesterol	88 mg/dL
Ferritin in serum	163.9 ng/mL
Iron in serum	92.91 ug/dL
Vitamin B12 (cyanocobalamin)	547.00 pg/mL
Vitamin D (25 OH-Vit D total)	35.60 ng/mL

Oral metoclopramide was discontinued, and after 48 hours of metoclopramide discontinuation, symptoms fully resolved, suggesting a drug-induced episode of RLS. Follow-up assessments at three and six months post-discontinuation revealed no recurrence of the symptoms.

## Discussion

This case is significant as it highlights a rare occurrence of oral metoclopramide-induced RLS in a patient with no history of RLS and normal laboratory parameters. The temporal association between the initiation of oral metoclopramide and the onset of RLS symptoms, followed by their resolution upon drug discontinuation, suggests a causative link.

The occurrence of RLS in the setting of oral metoclopramide therapy is rarely described in the current literature, whereas several cases are reported in the injectable form. Sieminski and Zemojtel previously published a case report about a 32-year-old woman with migraine who developed symptoms of RLS after receiving IV metoclopramide for migraine-associated vomiting. Management included treatment discontinuation with an intensive saline infusion, resulting in sustained resolution of symptoms [7]. In another publication, Moos and Hansen presented a case of akathisia (a condition similar to RLS) developed after preoperative administration of a single 10-mg dose of metoclopramide [6]. Similarly, Ibiloglu described an akathisia episode secondary to metoclopramide short use in a 56-year-old male with gastroesophageal reflux disease [8], while Qiu and Lim reported acute akathisia in a young woman who was given an IV bolus to treat her gastroenteritis [9]. Other cases of iatrogenic RLS secondary to selective serotonin reuptake inhibitors (e.g., citalopram) have been published [10].

Mechanistically, metoclopramide may elicit RLS as well as other movement disorders via its interference with extrapyramidal dopaminergic and cholinergic systems [6]. Hence, metoclopramide, through D2R activity, antagonizes dopamine action in the central nervous system, which may lead to disinhibition favoring acute episodes of movement disorders. Unilateral striatal injection of metoclopramide induced ipsiversive turning behavior in mice [11]. Moreover, the downregulation of D2R expression in white blood cells was shown to occur during RLS [12]. Pharmacological studies have shown a decrease in RLS symptoms with dopamine agonists and an opposing effect with dopamine agonists [3]. For instance, dyskinesia has been suggested to be an idiosyncratic response to metoclopramide [13], and it is likely that this may also be the case for RLS. This proposition is supported by the rarity of metoclopramide-related movement disorders [13], indicating that only a specific subgroup of the treated patients will develop an RLS-like reaction. In line with this, the presence of a family history of RLS in a first-degree relative of our patient strongly suggests potential familial susceptibility. This susceptibility could be accompanied by pre-existing intrinsic processes that favor the onset of episodes. Genetic polymorphism of CYP2D6 enzymes was shown to affect the pharmacokinetics of metoclopramide [14], which would possibly disrupt the drug’s pharmacodynamics (i.e., by increasing its bioavailability in the brain), ultimately exposing it to adverse reactions.

This case underlines the importance of considering medication-induced RLS in patients presenting with new-onset leg compulsive sensations and movement disorders, particularly in the context of recent medication initiation. Its particularity lies in the emergence of this adverse effect following the administration of metoclopramide in its oral form. Therefore, it is necessary to carefully assess the medication history of patients with new-onset movement disorders on metoclopramide and drugs with similar action. Once a metoclopramide-induced RLS-like reaction is suspected, treatment should be interrupted, and other dopamine antagonists may be avoided for a lifetime.

## Conclusions

This case report identifies RLS as a potential, yet less common, adverse effect of oral metoclopramide compared to its IV form. The reaction is notable for its benign and rapidly reversible nature after treatment discontinuation, highlighting the importance of monitoring RLS symptoms in patients prescribed oral metoclopramide. This finding emphasizes the variability of drug side effects between different administration routes and underscores the need for careful patient observation in pharmacotherapy. In a broader sense, this case serves as a reminder of the dynamic and sometimes unpredictable nature of drug reactions and the need for ongoing vigilance in pharmacotherapy.
